# Mechanism Design of Health Care Blockchain System Token Economy: Development Study Based on Simulated Real-World Scenarios

**DOI:** 10.2196/26802

**Published:** 2021-09-13

**Authors:** Se Young Jung, Taehyun Kim, Hyung Ju Hwang, Kyungpyo Hong

**Affiliations:** 1 Office of eHealth Research and Business Seoul National University Bundang Hospital Seongnam-si Republic of Korea; 2 Department of Mathematics Pohang University of Science and Technology Pohang-si Republic of Korea

**Keywords:** mechanism design, optimization, blockchain, token economy, eHealth, electronic health records, healthcare, economy, health records

## Abstract

**Background:**

Despite the fact that the adoption rate of electronic health records has increased dramatically among high-income nations, it is still difficult to properly disseminate personal health records. Token economy, through blockchain smart contracts, can better distribute personal health records by providing incentives to patients. However, there have been very few studies regarding the particular factors that should be considered when designing incentive mechanisms in blockchain.

**Objective:**

The aim of this paper is to provide 2 new mathematical models of token economy in real-world scenarios on health care blockchain platforms.

**Methods:**

First, roles were set for the health care blockchain platform and its token flow. Second, 2 scenarios were introduced: collecting life-log data for an incentive program at a life insurance company to motivate customers to exercise more and recruiting participants for clinical trials of anticancer drugs. In our 2 scenarios, we assumed that there were 3 stakeholders: participants, data recipients (companies), and data providers (health care organizations). We also assumed that the incentives are initially paid out to participants by data recipients, who are focused on minimizing economic and time costs by adapting mechanism design. This concept can be seen as a part of game theory, since the willingness-to-pay of data recipients is important in maintaining the blockchain token economy. In both scenarios, the recruiting company can change the expected recruitment time and number of participants. Suppose a company considers the recruitment time to be more important than the number of participants and rewards. In that case, the company can increase the time weight and adjust cost. When the reward parameter is fixed, the corresponding expected recruitment time can be obtained. Among the reward and time pairs, the pair that minimizes the company’s cost was chosen. Finally, the optimized results were compared with the simulations and analyzed accordingly.

**Results:**

To minimize the company’s costs, reward–time pairs were first collected. It was observed that the expected recruitment time decreased as rewards grew, while the rewards decreased as time cost grew. Therefore, the cost was represented by a convex curve, which made it possible to obtain a minimum—an optimal point—for both scenarios. Through sensitivity analysis, we observed that, as the time weight increased, the optimized reward increased, while the optimized time decreased. Moreover, as the number of participants increased, the optimization reward and time also increased.

**Conclusions:**

In this study, we were able to model the incentive mechanism of blockchain based on a mechanism design that recruits participants through a health care blockchain platform. This study presents a basic approach to incentive modeling in personal health records, demonstrating how health care organizations and funding companies can motivate one another to join the platform.

## Introduction

Precision medicine aims to define diseases at a higher resolution using genomic data, electronic health records, and life-log data by providing new therapies to each targeted subgroup [1,2]. Electronic health records and life-log data from personal health records are crucial to capture phenotypic information in hospitals and in everyday life in order to deliver precision medicine to health care consumers. This is one of the major reasons why even high-income nations have been struggling over the past couple decades to properly disseminate electronic health records. For instance, the United States implemented the Health Information Technology for Economic and Clinical Health Act in 2009 to provide electronic health records throughout the country [3]. By 2017, 96% of general medical and surgical hospitals, 87% of children’s hospitals, and 59% of acute long-term care hospitals in the United States had adopted certified electronic health records [4,5]. South Korea has also tried to disseminate electronic health records since early 2000. The Health Insurance Review and Assessment Service announced that 93.6% of hospitals and 91.6% of private clinics in South Korea utilized electronic health records as of 2017 [6]. Even though the adoption rate of electronic health records has increased dramatically throughout many countries, they are still struggling to find solutions to properly disseminating personal health records. Compared to the implementation and use of electronic health records in health care organizations, the implementation of personal health records, for the collection of life-log data through patient participation, still lags due to challenges related to security, privacy, interoperability, and data quality [7-9]. The implementation of personal health records faces other issues as well, including a lack of auditability, legal risk, health care policies, and data accuracy.

As such, health care blockchain has been implemented to solve these various problems. Blockchain is known as a distributed ledger technology—it records given information into small chunks of data sets called *blocks*, and if recorded data are valid, the blocks are, in turn, chained with a consensus protocol. The data are stored in a peer-to-peer system-based distributed storage environment, which does not permit anyone to arbitrarily modify it, because anyone can determine when a data change occurs [10]. Therefore, applying blockchain technology to personal health records strengthens the integrity and security of the clinical data stored within [11]. In addition, it is expected that automation features, such as smart contracts, will reduce both cost and time in managing patient participation through the dynamic consensus system [12,13]. Such an appropriately designed blockchain token economy can help in devising strategies to find the benefits of participating in clinical data sharing and to ensure their fair distribution among multiple stakeholders [14]. Furthermore, blockchain can improve the auditability of transferred health care records with secure privacy, help authenticate participants in health information exchange networks with distributed identification, and boost patient participation in the platform by providing incentives according to active participation and adherence to the system [15].

For the 2 main advantages of blockchain—namely auditability and identification—many proof-of-concept studies have been conducted [11,16-18], yet few studies have explored the incentive mechanism of blockchain with respect to real-world cases, in which incentives are crucial for recruitment and attention [19].

To examine the effectiveness of incentivization, one study [20] designed a token economy to encourage adherence to activities of daily living—bathing, physical activity, and oral hygiene—to reduce the risk of bloodstream infections, oral complications, and deconditioning in hematopoietic stem cell transplant for pediatric patients. Activities of daily living adherence rate increased from 0.51 to 2.5 after implementing the token economy [20]. In other studies [21-23], the preferred incentive methods were free parking, modest financial compensation, food coupons, guitar lessons, transportation, and donations to charity. Though £100 (approximately US $137.51) as an incentive was effective when recruiting older adult patients in a clinical trial [24], an amount of US $2.00 as an incentive was not effective [25]. This indicates that the amount of monetary compensation is important. Incentivizing also proved to be effective for patient adherence to smoking cessation, diet, and digital therapeutic programs [26-29]. Some questions have arisen—How about giving incentives multiple times? How much should we compensate for participants’ effort? How long can we collect patients’ information on the blockchain platform? Is it possible that the shorter the collecting time, the bigger the incentives become? In digital therapeutics, one main strategy in behavior modification is to make a contingency plan to address poor adherence, and monetary reward seems to be the most effective way for active engagement [30].

Mathematical studies have been performed to model incentivization for blockchain. For decentralization, designing protocols that make it difficult to tamper with transactions is essential, and a mathematical approach to designing blockchain protocol that achieves secure information exchange has been examined [31,32]. In addition, the analysis of players' mining strategies was conducted on blockchain using game theory [32,33]. Classical mechanism design theories have been examined on some apps, such as Auction [34]. However, there has been no research linking apps in the blockchain system to mechanism design, which is considered to be representative token design theory for blockchain [35-38].

Why is a new theoretical foundation needed for health care blockchain cryptoeconomics? The purpose of cryptoeconomics is to create internet services, but why do we need new theories and methodologies? In short, it is because the design of decentralized networks is completely different from that of traditional business/service planning. Existing business planning is given certain rules (market conditions) and aims to ensure that companies make the best choices to maximize profits; however, the design of a decentralized network aims for the opposite. Assuming that each entity acts strategically and selfishly, the design of a decentralized network makes rules to achieve desired results.

Despite the pressing need for further research on the cryptoeconomics of health care blockchain, there has been a lack of research on token economy design to induce patient engagement using health care blockchain automated incentive programs. Thus, we aimed to explore parameters to consider when designing incentive programs that can be embedded in the smart contract of blockchain by using experiments based on 2 representative virtual scenarios with mechanism design.

## Methods

### Methodological Background

Blockchain-based networks have no central principal. Therefore, we need a system that uses tokens (as the medium) and market principles to help individuals grow the network even if they act in pursuit of their own interests. This is called the *Token Model*, which serves as the invisible hand in decentralized networks [39].

**Figure 1:**
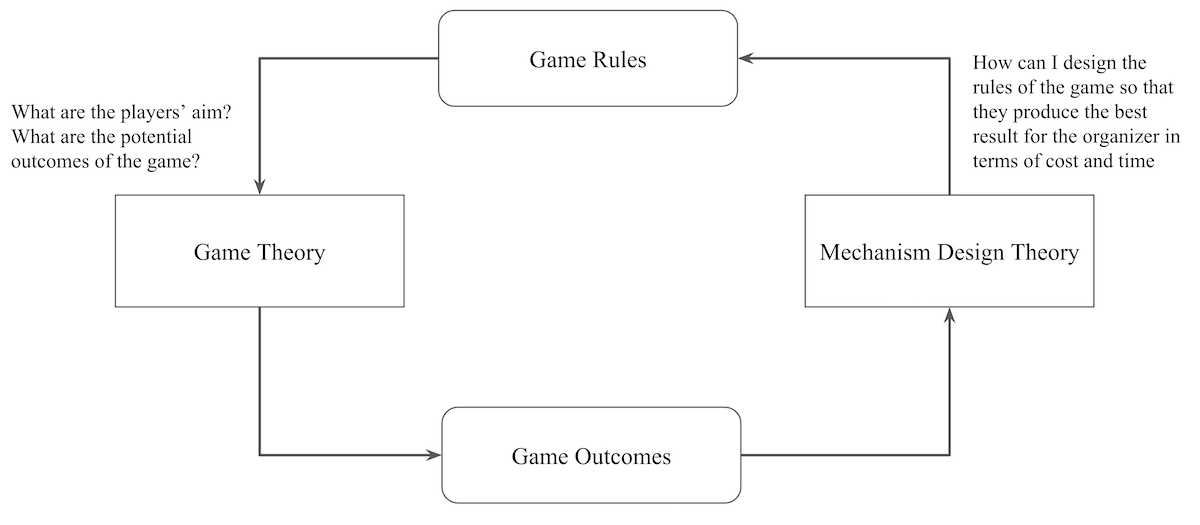
Relationship between game theory and mechanism design theory.

To design the token economy, we considered 2 well-known economic theories: game theory and mechanism design. In game theory, existing business planning is given rules (market conditions) and aims to ensure that companies make the best choices to maximize their profits. The theory examines the best strategy in a given game. It explains how to draw conclusions in situations in which several rational decision-makers compete to win over one another. It is called a *game* because it is reminiscent of an actual game with competitors using strategies to win [40]. The relationship between game theory and mechanism design is as described in Figure 1. The organizer of recruitment is able to set the game rules in order to achieve intended goals in terms of cost and time in our scenarios (Figure 1 [41]).

However, the design of a decentralized network is the reverse. Mechanism design uses an engineering approach in which designers act rationally toward desired goals in a strategic environment by applying economic mechanisms and incentives to design strategies. It is also called *reverse game theory* because it starts at the end of the game and moves backward [34].

With the principle of mechanism design, we can reduce cost caused by trial and error and provide a platform for solving real-life problems as a theoretical model. Combined with blockchain, mechanism design has been adopted in various types of research. The auction is a representative example of mechanism design in a blockchain environment with randomness and information disclosure [36]. Based on this possibility, a previous study [35] provided the framework that analyzes the blockchain protocol using mechanism design and game theory. These studies [35,36] suggested that mechanism design can be applied to the blockchain system, especially to the health care blockchain economy, thereby resulting in the development of a basic token economy. We wanted to make ground truth models for the token economy of patient participation and data provision in personal health records. First, we assumed a basic scenario with essential variables—recruiting healthy participants for a vitality program that aims to reduce weight. The vitality program is a technology-based wellness program that is included with most life insurance policies, to support and reward healthy habits [42,43]. Second, we validated model robustness with another hypothetical scenario for validation—recruiting participants for clinical trials of anticancer drugs.

### Assumption of Roles on a Blockchain Platform

When recruiting participants to a vitality program in the real world, a life insurance company provides incentives to those involved in the program. This mechanism is easily applied to the blockchain using smart contracts. In addition, the blockchain protects personal health information and is able to conduct universal recruitment through the app; therefore, efficient and safe recruitment of participants can be carried out through health care blockchain.

Meanwhile, the compensation that companies provide to participants on the blockchain is cryptocurrency. Therefore, it is essential to discuss how to calculate the value of this cryptocurrency, which can be performed in many ways. The value of bitcoin is determined by the free market. Suppose a vitality program participant is recruited on a blockchain; the life insurance company must have a certain amount of cryptocurrency. A participant who received the cryptocurrency must be able to exchange it for their benefit. In a conventional blockchain, operators obtain cryptocurrency in exchange for maintaining a blockchain system. However, unless the life insurance company participates in the blockchain operation, it must purchase the cryptocurrency from another party to secure a certain amount of it. In addition, assuming that the value of this cryptocurrency changes, there could be some concern that the total amount of money in the blockchain system would become negative. To solve these problems, we define a concept called *a currency exchange*, which assumes that a certain amount of cryptocurrency can be purchased or exchanged in a certain amount of fiat currency. The advantage of this method is that the total amount of cryptocurrency in the entire blockchain system, and the fiat value of cryptocurrency does not fall below zero, which helps the health care blockchain operate in a stable manner.

Several members exist in a blockchain system with a currency exchange. First, there is a blockchain operation party that operates the blockchain and acts as a currency exchange. In this study, it is assumed that the blockchain operating party does not affect the token economy because the party only generates cryptocurrency, and the value of cryptocurrency is fixed via the exchange. Second, there is a data provider that has patients’ health information. Members who store personal health information include entities such as hospitals or genetic companies. They receive cryptocurrency from members who request information when there is a transaction of the information they store. The reason for receiving the cryptocurrency is the cost of storing the information. The third party is a user on the medical information platform. Users can be patients requesting their genomic information, healthy people uploading their life-log data, or life insurance companies wanting to recruit participants. In this study, we assume a life insurance company prepares a vitality program from the perspective of mechanism design by recruiting participants and finding out how long the recruitment period will be, depending on the amount of the incentive.

### Scenarios

#### Scenario for the Development of the Model

**Figure 2:**
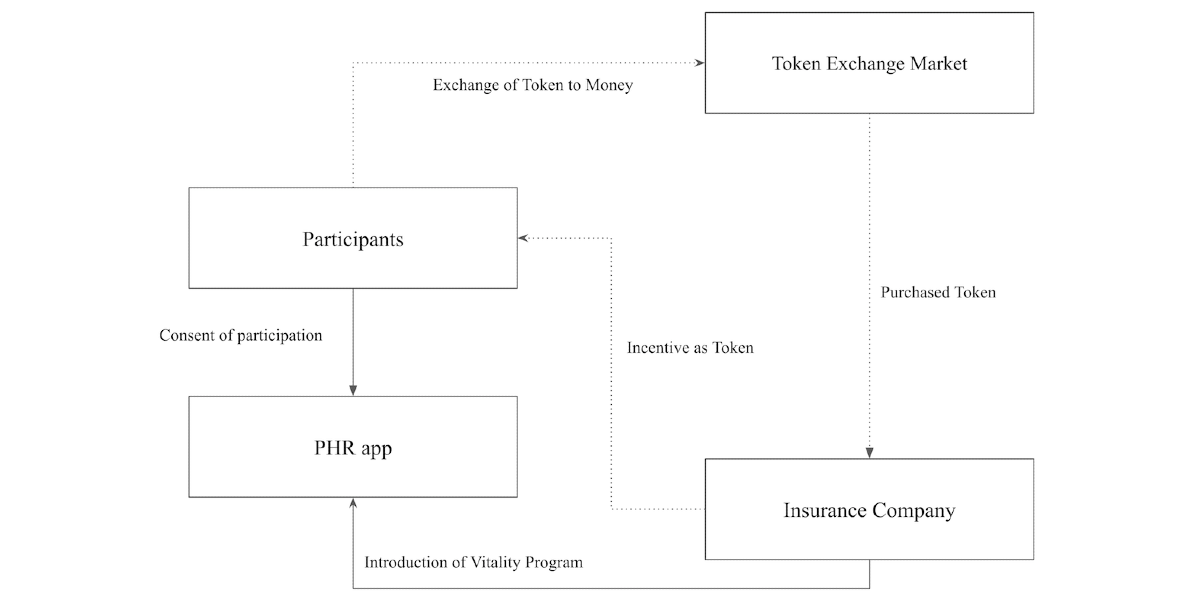
Basic scenario. PHR: personal health record.

Figure 2 shows a brief process of the basic scenario. The insurance company uploads the following program information through mobile phone apps regarding the collection of life-log data: (1) the institutions that conducts a vitality program; (2) duration of program operation; and (3) token rewards for participating in the program. Based on this information, participants will decide whether to take part in the program. If they decide to participate, they send requests through the app. The vitality program organizer will track participation status until a certain number of participants are obtained. The number of participants obtained here is different from the number of *N* desired by the organizer because the organizer needs to select participants based on their demographic information so that the sample is as unbiased as possible. If participants with certain socioeconomic statuses are enrolled more, the result of the intervention and analysis on effects of the program can be biased. The number *N* is set to greater than the necessary number of participants for the program because some enrollees could be dropped after adjustment for socioeconomic status. The vitality program organizer will pay participants who agree after screening tokens through the app.

#### Scenario for the Validation of the Model

**Figure 3:**
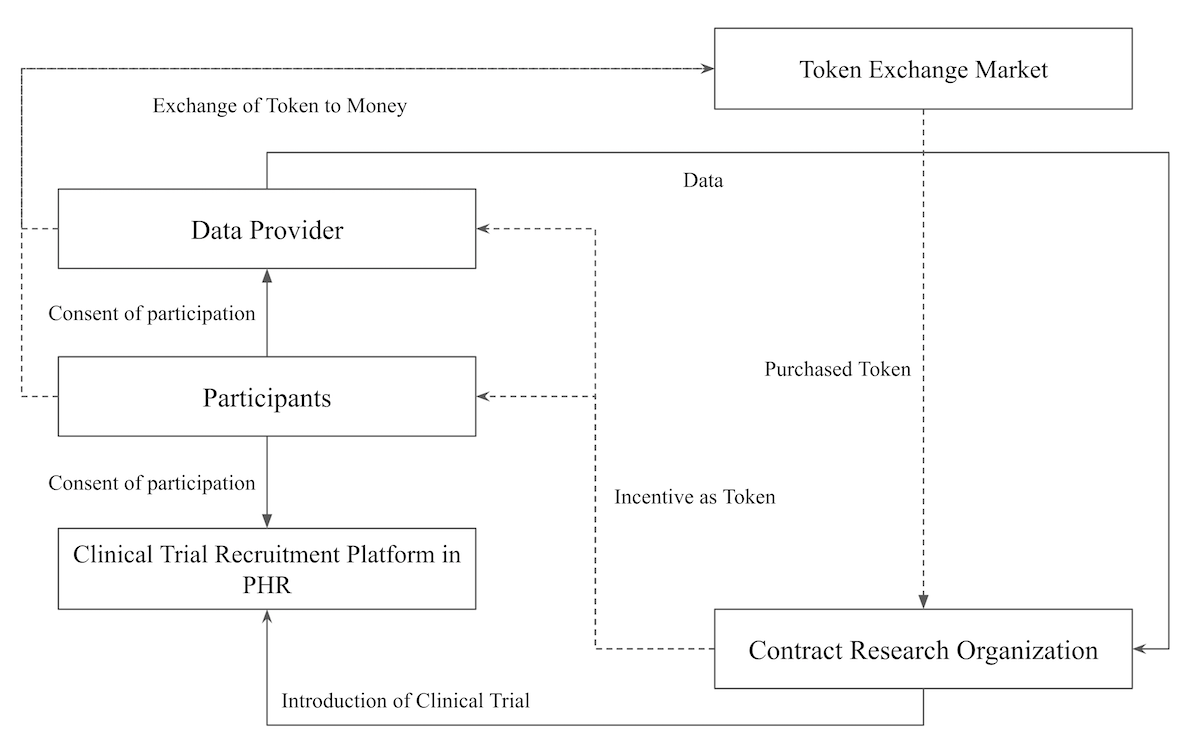
Validation scenario. PHR: personal health record.

We established the validation scenario by modifying the basic scenario (Figure 3). In the validation scenario, the research organization searches for participants who have specific gene mutations on the blockchain network for clinical trials of anticancer drugs. For convenience, participants who satisfy all the information required by the researcher are described as group A. Potential participants who do not have necessary clinical information are described as group B. The researcher sends a request for consent to both group A and group B.

The participants will decide to participate in the research based on the opinion of doctors who are treating their cancer. In our study, we assumed that the smaller the amount of clinical information that participants are required to send, the higher the probability of consent. This is based on the general assumption that the more information participants have to provide, the more careful they are because normally people care about privacy and security.

For example, if participant X in group A agrees to consent, the institution storing the data of participant X shares the data with the research company and the institution obtains cryptocurrency compensation. In the validation scenario, the companies transfer tokens to data providers that have clinical information for candidate patients in exchange for compensation for storing the data, which is different from the basic scenario in that data providers are added in the token flow

#### Mathematical Modeling of the Scenario

Mathematical models for both scenarios were constructed; a methodology that minimizes the cost of the organizers was modeled. The essential members were participants and organizers. In both scenarios, the reward was the main parameter that affects participation—we assumed that the higher the reward, the greater the probability of consent. In the case of an organizer, an assumption is needed about which information can be obtained from the participants and the organizers’ cost policies. The cost was divided into 2 categories: reward-related parameters and time cost–related parameters.

#### Cost Function of a Life Insurance Company

We assume that the organizer's cost function is divided into 2 parts—reward and cost. Rewards are provided to participants; therefore, the reward portion is multiplied by the total number of participants. The time is also included in the time cost, and because the rewards and time are different in units, it is necessary to balance both parts by giving one part a weight. In our model, we give a weight to the time cost part (the time weight). The time weight may vary in different recruitment programs. A higher time weight means that an organizer values recruitment time more and wants to recruit participants quickly. We express the cost function as *cost* = *reward* × *N* + *t* × α.

When minimizing the cost, if the time weight α is small, cost will be largely dependent on the reward. The unit of time *t*, where *t* is a natural number, is assumed to be day for convenience.

In addition, the recruiting time calculation method should be defined. The organizer sends consent requests to those who meet the requirements, and it is assumed that each participant has a probability of consent. Under the assumption, the time taken for recruitment is calculated as follows. On day 1, the organizer sends consent requests to the people in the participant pool. Some participants agree based on their probability, and others disagree. If the number of people who agree does not satisfy the required number of recruits, on day 2, the organizer will send the consent request again to those who did not agree. This process is repeated to calculate the recruiting time. Recall that the probability of consent to participate in research is dependent upon the reward, and the higher the reward, the greater the probability of consent. Increasing the probability of consent means it will be easier to recruit participants, and the time *t* for recruitment completion will be reduced.

#### Modeling the Basic Scenario

We assumed that participants can access information about how long the program lasts and the reliability of the insurance company. They also consider inconvenience that may be caused by joining the program.

The welfare of a participant is separated into 2 parts: (1) monetary value and (2) labor. In order to have unbiased results, the socioeconomic status of participants should be considered. The lower the status, the more significant impact on the monetary part, which results in biased recruitment; therefore, we introduced a concave function to represent monetary welfare. Participants have information about their socioeconomic positions, the degree of annoyance for recording tasks, and the reliability of life insurance companies, which are not disclosed to the research companies. Let the socioeconomic status of the *i*th participant be *SP_i_*, let the degree of annoyance be *b_i_*, and let the reliability of the company be *R_i_*. Life insurance companies can set the program period *T* and compensation rewards for the programs, which are communicated to participants through apps.

The participant's welfare is defined using the square root (which is a concave function), which means the higher *SP_i_*, the lower the incremental increase in total welfare of participants. The welfare function of the *i*th participant is



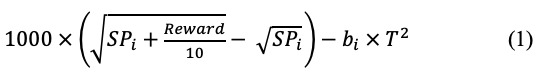



The former term is the weight given to the welfare according to the socioeconomic status, and the latter term is the fatigue of continuously uploading the log by multiplying the basic quadratic function for *T* by weight. Therefore, for participants, a sufficient condition for participation is that the welfare function is positive.

Socioeconomic status is likely to be biased because it provides compensation for participation. Therefore, at initial recruitment, a certain number of the desired population is selected to ensure that socioeconomic status is as evenly matched as possible. In labor-related welfare, fatigue builds up faster over time, and each person’s degree of annoyance is different. Hence, the labor-related cost is proportional to the degree of annoyance and the square of the duration.

In addition to consideration of financial benefits, the probability of participating in the program was introduced (to consider the psychology of real-world participants). The probability will be large when the reward is large, the reliability of the organizer is high, and the socioeconomic status is low.

Assuming that the welfare function of the *i*th participant is positive, the probability that the *i*th participant will consent is calculated based on the following assumptions: (1) the higher the rewards, the higher the confidence in the research company; and (2) the lower the socioeconomic level of the participant, the higher the probability of participation. Thus, the probability is calculated as



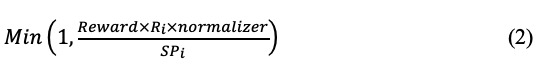



The normalizer is a buffer weight to allow the rewards to grow. If the right term of equation 2 is greater than 1 for most of *i*, the simulation becomes meaningless; thus, the right term is changed to a slightly more meaningful simulation by multiplying the normalizer by less than 1. In the basic scenario, *SP_i_*=5-10, *b_i_*=0.05-0.1, *R_i_*=0.5-0.7, and *normalizer*=200.

#### Modeling the Validation Scenario

We modified the previous assumptions and added an intermediate data provider in the validation scenario to ensure that the model is robust after we change variables.

For data providers, assumptions are not necessary because there is no separate strategy available; however, there is a higher chance of making a profit in proportion to the amount of information held by the data providers. A research company can define its cost function, which sets the compensation and time weight per participant. Participants must decide whether to agree or not for the consent request they received. Theoretically, participants decide to agree when their economic gains are greater than 0. In real life, however, other factors play a role in determining whether a patient will be able to participate in clinical trials. Thus, we assume that the agreement probability is based on the expert advice which is provided through the blockchain platform. Normally, patients get expert opinions from their oncologists. If *k* is the number or range of treatment options available, the smaller the *k*, the higher the probability of consent. Utilizing a sigmoid function, the probability of consent has a value between 0 and 1.

Participants in research must disclose whether their genes have been tested, genotype (if they have been tested), and which data provider is storing their data. Moreover, some information on the medical blockchain is open to companies: (1) whether one’s genes have been tested and (2) genotype. A few properties are needed to describe this. First, *DP_i_* is defined to determine whether the genetic data of the *i*th participant are stored by the data provider. A *DP_i_* of 0 indicates that there are no genomic data, and a value greater than *n* means that information is stored by the *n*th data provider. The participant's genotype is defined as *Type_i_*. If *Type_i_* is 0, it means that the genotype is not known because it has not examined, and *DP_i_* has a value of 0. If *Type_i_* is *n* with a value greater than 0, the dielectric means it is *n*th type.

In the basic scenario, the probability of consent of participants is the expert advice. The greater the rewards, the smaller the number of protocols available to participants and the greater the expert advice. The probability can be defined as a simple product in the basic scenario, but in the validation scenarios, the sigmoid function is used,



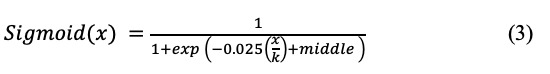



The sigmoid function converts a real value to a value between 0 and 1. Therefore, this value has the advantage of being used directly as a probability. When *x* is 0, the probability should be close to 0. To shift the sigmoid function to the right, *middle* should be introduced. In the validation scenario, *SP_i_*=0-3, *k*=1-5, *DP_i_*=1-2, and *middle*=150.

### Optimization Formulation

We chose the convex optimization model. For each scenario, we suppose that the expected recruitment time can be found when the clinical trial is given a reward. For each reward and time of completion of the recruitment pair, we obtain the cost of applying the pair. The reward and time *t* at the lowest cost point will be the optimization values.

To find the expected recruitment time for a given reward amount, the probability of each participant agreeing to participate in the clinical trial is determined. Then using Bernoulli implementation, we obtain the expected number of participants that agreed until *N* days. Suppose that the probability that the *i*th participant agrees is *q_i_*. In that case, whether one will eventually agree by the maximum *N* days is the same as the Bernoulli implementation. The probability of agreeing on the first day is *q_i_*, the probability of agreeing on the first day and agreeing on the second day is (1 – *q_i_*)*q_i_*, and the probability of agreeing on the third day is (1 – *q_i_*)^2^*q_i_*. When this is implemented by the *j*th day, the expected number of participants that agreed until *j* days can be obtained as



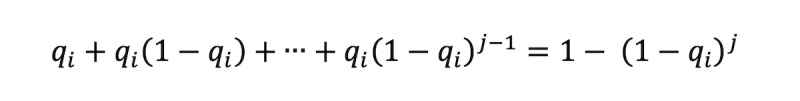



Therefore, if these expectations are added for all *i*, the expected number of participants until *n* days is obtained. When rewards and *n* are given, the expected number of participants can be obtained, and if *n* is obtained, this value *n* is the expected completion of the recruitment.

Aggregating the expected values, we determine the expected number of participants upon *N* days. When we find the minimal *N* that the expected number of participants exceeds the company targeted number of participants, then we obtain the reward and the minimal *N* pair. We put the pairs into the cost function, and find (reward, *N*, cost) pair that the cost is minimal.

## Results

### Optimization

**Figure 4:**
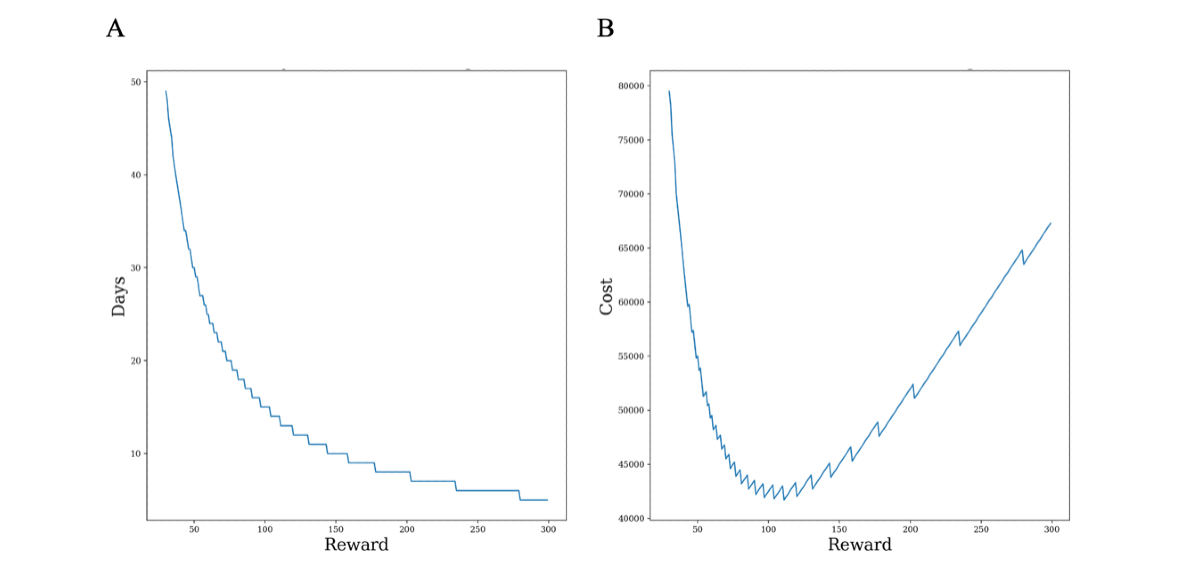
(A) Time–reward and (B) cost–reward trade-offs in the basic scenario when the time weight is equal to 1500.

**Figure 5:**
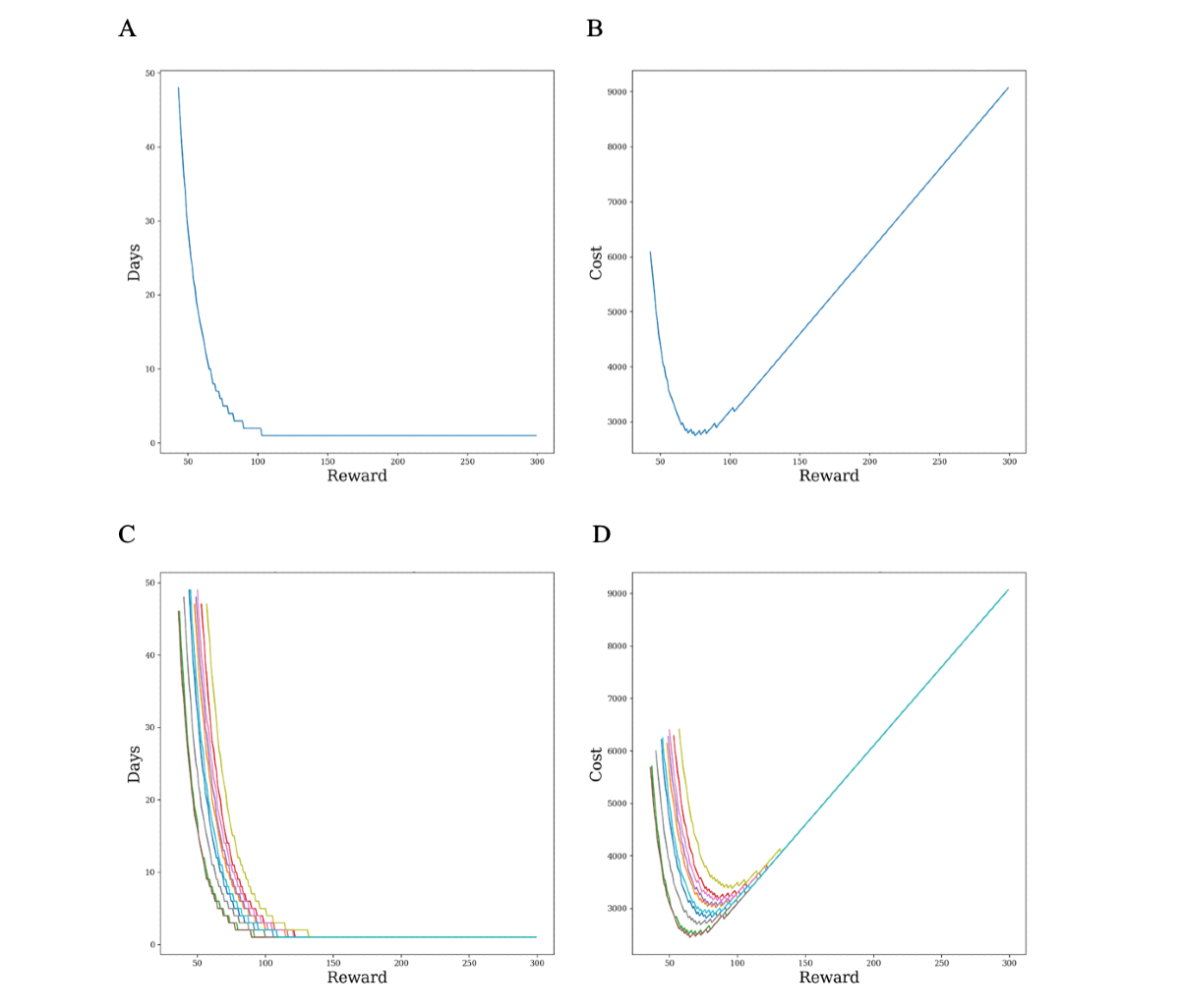
Single-case (A) time–reward and (B) cost–reward and multiple-case (C) time–reward and (D) cost–reward trade-offs in the validation scenario when the time weight is equal to 100.

The reward affects the probability of consent of each participant, and this probability of consent affects the expected recruitment period *t*. Note that the expected recruitment period is a function of the reward. Therefore, rewards and expected recruitment period pairs can be obtained. We can make a tuple by considering the cost value of these pairs applied to the cost function. Thus, the relationship of *t* with the reward and the cost relationship with the reward can be obtained. Figure 4 shows the relationship when the company's time weight is 1500 in the basic scenario, and Figure 5 shows the relationship in the validation scenario in which the company's time weight is 100. Because the basic scenario assumes a company cannot access some of the participant's information, we should use expected values of distributions when we calculate the expected recruitment time. Therefore, even if participants' information is changed through initialization, the graphs are unified into one. On the other hand, the validation scenario allows the company to access all of the participant's information, which means that we should consider each participant’s information when we calculate the expected recruitment time. Therefore, as the participant's information changes, the graph changes accordingly. Figure 5C and Figure 5D show 10 initializations.

In Figures 4 and 5, the graphs on the left are the relationships between rewards and expected recruitment periods, and the graphs on the right are the relationship between rewards and costs. The smaller the rewards, the larger the expected recruitment period, and vice versa. The right graph that puts the rewards and expected recruitment period in the cost function forms a convex curve. The left side of the shape is high because the time cost has grown, and the right side is large because the reward cost affects the entire cost. Therefore, the left part shows a similar appearance as the time decreases dramatically. The right side becomes a linear graph for the reward because the time cost becomes very small. The middle part of the trade-off between these 2 will be the point where the cost is minimized, and the reward value and the expected recruitment period will be reward* and t*.

### Comparison With Simulation

**Figure 6:**
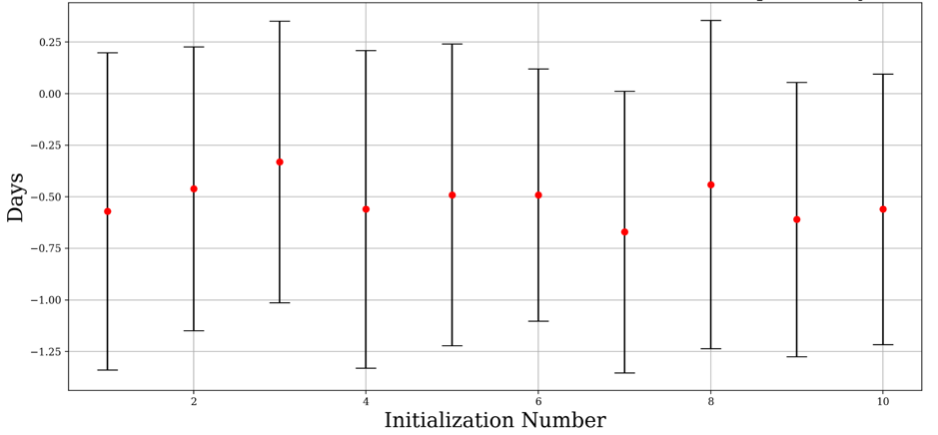
Basic scenario mean values and standard deviation of errors (expected days=13).

We compared the simulation results with optimization results. Since reward* is an independent variable, it is appropriate to compare dependent variable t*. In order to obtain robust results, we conducted repetitive experiments by changing the variables of participants randomly. For each initialization, we simulated 100 times to get the experimental results. Then, we calculate the error between 100 experimental results and recruitment time, t*. Finally, we obtained the mean and standard deviation of those values. In the basic scenario, the simulation values are concentrated at the optimized values (Figure 6, Table 1).

**Figure 7:**
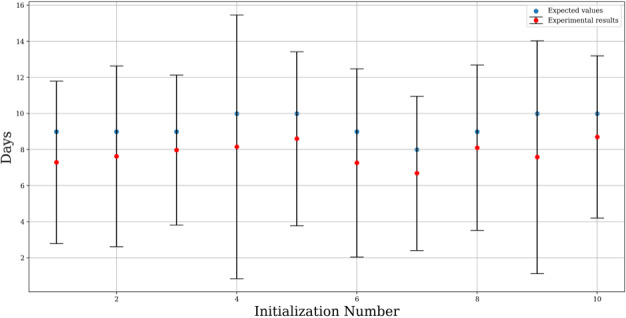
Validation scenario mean values and standard deviation of errors.

Figure 7 and Table 2 show differences between optimized results up to a maximum of 2 days for the validation scenario.

**Table 1:** Basic scenario.

Experiment	Error, mean (SD)
1	–0.57 (0.77)
2	–0.46 (0.69)
3	–0.33 (0.68)
4	–0.56 (0.77)
5	–0.49 (0.73)
6	–0.49 (0.61)
7	–0.67 (0.68)
8	–0.44 (0.80)
9	–0.61 (0.67)
10	–0.56 (0.66)

**Table 2:** Validation scenario.

Experiment	Expected days, n	Error, mean (SD)
1	9	–1.70 (4.51)
2	9	–1.37 (5.01)
3	9	–1.02 (4.16)
4	10	–1.84 (7.31)
5	10	–1.39 (4.82)
6	9	–1.73 (5.22)
7	8	–1.31 (4.28)
8	9	–0.89 (4.59)
9	10	–2.41 (6.45)
10	10	–1.30 (4.50)

### Sensitivity Analysis

The company can adjust the number of participants *N* and time weight α; therefore, we analyzed the sensitivity of t* as *N* and α change. For 1% increments of *N,* recruitment time increases 1.54% and 2.22% in the basic and validation scenarios, respectively. If the time weight is increased by 1%, the recruitment time is decreased by 1.54% and 1.67% in the basic and validation scenarios, respectively, which means this model is in line with our general knowledge and provides a guideline for designing recruitment of participants.

## Discussion

### Principal Findings

In this paper, we propose 2 token economy scenarios of health care blockchain with mechanism design. We set basic components in each scenario, which were the number of participants and recruitment time, constructed mathematical models to explain the 2 scenarios, and simulated changes in recruitment time and the number of expected participants. Through mechanism design, we demonstrated that the recruiter is able to set a desired and expected number of participants and recruitment time by adjusting the amount of incentive. This study is the first, to the best of our knowledge, to apply mechanism design to health care blockchain for real-world problems.

In classic game theory, designers of the game are not able to determine expected results of games quantitatively. They can expect rational participants to compete with each other for the best results by assuming that participants are reasonable, therefore, act to maximize their profits; however, it is not realistic to apply this assumption directly in the health care blockchain with token economy because it is crucial for a funder of health care blockchain to know expected time, cost, and the number of recruited participants.

In our models that employ mechanism design, the recruiter can set a quantitative outcome of programs by adjusting the relationship of number of participants, recruitment time, and rewards. In addition to the predictability of outcomes, the blockchain system can provide participants more secure environments with immutability of the system, although the system is more open to the public compared to conventional ways of recruiting participants.

Traditionally, the issue of whether direct incentives for health care participants are acceptable has been debated because direct incentives can bias medical research and treatment outcomes [44]. From a medical standpoint, there is less of an ethical problem with direct incentives for healthy people [45], such as those modeled in the basic scenario. In the validation scenario, there may be a concern with direct incentives to participants within the blockchain. However, we assumed a circumstance that allows direct incentives for the participation of clinical trials in the future because patients are selected randomly as participants on a blockchain system, and we assumed that direct incentives can be allowed to increase recruitment and attention rates in clinical trials.

From a mathematical perspective, information about each participant is different depending on whether recruiting companies have access to it or not. While recruiting healthy individuals, we assumed that information about each participant is unobtainable by the company because they usually do not need strict inclusion criteria, which means that they can exclude some of applicants after closing the recruitment. When collecting life-log data, the degree of annoyance for each participant over a long period is unobtainable and we can only see the distribution for the degree of annoyance. In this study, this distribution is assumed to be a uniform distribution. In this case, a clinical trial company uses the expected value of the distribution (ie, the uniform distribution's average value). Meanwhile, in the validation scenario, each participant should provide gene data to the research company. From the gene data, the company can infer the probability of agreement of each participants and can adjust model to fit real-world data.

Recruiting participants is one of the most difficult parts of conducting a clinical research or trial. However, the token economy, through mathematical modeling and simulation techniques proposed in this study, will enable companies to obtain important insight into whether they can recruit participants within the desired period by setting appropriate rewards for recruiting participants. Moreover, due to difficulties in recruiting participants suitable for clinical trials, companies may make a request to hospitals for patients information. However, hospitals do not feel the need and reason to provide data they hold, or even if hospitals are willing to provide data, the consent process for the use of patient data is complicated, and many patients are concerned about privacy and security [46]. Therefore, blockchain token economy can be applied to appropriately compensate entities participating in the blockchain data sharing platform, thereby reducing gaps between the needs of companies, hospitals, and participants (patients) with respect to data utilization, with high security and privacy, enabling efficient recruitment of participants through user-centered participation. This study is meaningful because we were able to make rational models that can be used as a starting point for designing health care blockchain for patient recruitment.

### Limitations and Future Research

Our assumptions did not reflect complex circumstances for recruiting participants in the real world. Thus, if the results of our models are different from the expectations of recruiting company, recruitment would not be successful. Models with basic mandatory requirements such as recruitment time, amount of incentives, and number of participants, and without complicated assumptions, can be useful for other researchers as a starting point of their own modeling—they can use our framework as a basic scenario to design their own sophisticated token design in health care blockchain. Another limitation is that comparison and analysis with actual data could not be carried out to provide a more robust framework.

### Conclusion

There have been few business models adopting blockchain technology in health care. Token economy of blockchain can be a powerful driver by incentivizing health care consumers with an immutably trackable token transaction system. This research is a starting point of designing the token economy in real-world health care settings; we modeled 2 possible scenarios, optimized the cost of the company, and compared the results with simulation data. This study can be used as a basis for designing an incentive system for companies recruiting clinical trial participants or health care program participants within a set period of time with a blockchain-based patient recruitment platform.
